# Rapidly progressive hip disease—A rare entity in Korean population

**DOI:** 10.1016/j.ijscr.2018.11.055

**Published:** 2018-11-24

**Authors:** Rohan Bhimani, Preeti Singh, Fardeen Bhimani

**Affiliations:** a Department of Orthopaedics, Hinduja Healthcare Surgicals, 11th Road, Khar (West), Mumbai, 400052, India; b Department of Orthopaedics, Osmania General Hospital, Hyderabad, 500012, India; c Department of Orthopaedics, Bharati Hospital, Pune, 411043, India

**Keywords:** RPHD, Rapidly destructive osteoarthritis, Arthritis, Rheumatoid arthritis

## Abstract

**Introduction:**

The pathophysiology of rapidly progressive hip disease is still unclear. It is a rare subset of osteoarthritis that mainly affects elderly women and presents with new- onset severe hip pain and dysfunction. It is assumed to be triggered by extremely rapid osteoarthritic changes resulting in impaction of the femoral head into the acetabulum, with successive osteonecrosis and insufficiency of the femoral head.

**Presentation of the case:**

A 62- year-old woman reported of right hip pain which succeeded an acetabular fracture for which open reduction and fixation was done. Initial radiographs, post fixation, showed no obvious abnormality. After 2 months of conservative therapy for her right hip pain, radiographs showed joint space reduction and subchondral bone loss. T1 MRI images revealed marrow oedema pattern and depression of the articular surface of the femoral head.

**Discussion:**

Differential diagnosis includes conditions which potentially lead to rapid hip destruction, such as metabolic bone diseases, autoimmune inflammatory arthritis and classical osteonecrosis. In patients with rapid worsening of hip symptoms successive X-rays and a high degree of clinical suspicion towards RPHD is essential to avoid extensive joint destruction and to facilitate better arthroplasty outcomes.

**Conclusion:**

The authors propose that this case represent an uncommon subset of osteoarthritis. Regular review, both clinically and radiologically, is required to assess speed of progression and to prevent rapid loss of bone stock without the surgeon being aware.

## Highlights

• The purpose of our case report is to make clinicians in Orthopaedic and Rheumatology clinics aware of the clinical and radiological features of this rare hip disease, which could be frequently confused with other more commonly acknowledged hip arthropathies.
• Such cases are frequently seen in individuals of European decent, but sparingly seen in those of American, Japanese and Middle Eastern decent.
• To author’s knowledge, it has never been reported in Korean population in the literature.

## 1 Introduction

Rapidly progressive Hip Disease (RPHD) also known as Rapidly Destructive Osteoarthritis of the Hip joint is a rare disorder of unknown causative factor. It is a completely different entity compared to osteonecrosis of the femoral head, and it results in fast deterioration of both the femoral head and acetabulum and finally concludes by disappearance of the femoral head [[1], [2], [3], [4]]. It occurs within months after initiation of the symptoms and is commonly seen in elderly females, in a hip joint that initially appears to be normal [[1], [2], [3], [4], [5], [6], [7]]. The disease has been reported frequently in the European literature [1,3,[8], [9], [10]]. But is only sparsely reported in American, Japanese and Gulf countries [2,[4], [5], [6], [7],^11^]. To the Authors’ knowledge, this disease has never been reported in the Korean population. The purpose of our case report is to make clinicians in Orthopaedic and Rheumatology clinics aware of the clinical and radiological features of this rare hip disease, which can be frequently confused with other more commonly acknowledged hip arthropathies.

## 2 Methodology

The work has been reported in line with SCARE criteria [12].

### 2.1 Case report

**Fig. 1:**
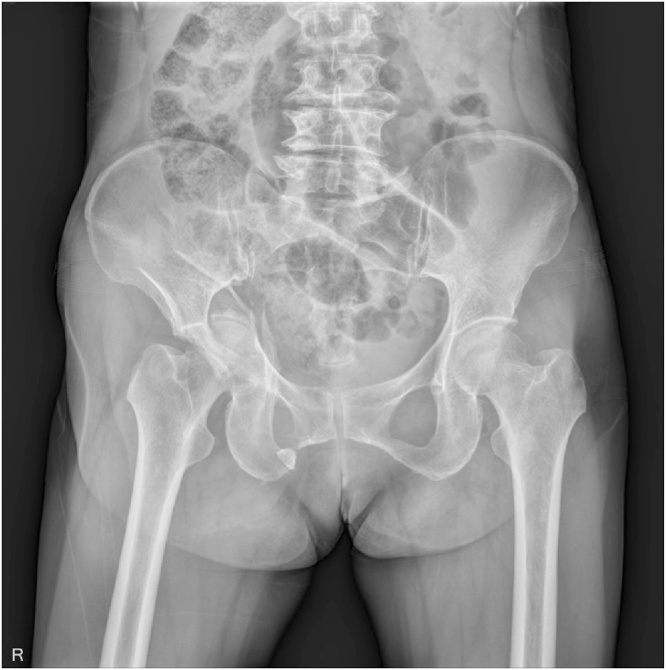
Both column right acetabular fracture involving the quadrilateral plate, superior and inferior pubic rami. Head of the femur appears to be normal.

A 62-year-old Korean woman presented to the Emergency Department after a road traffic accident. On clinical and radiological evaluation, patient was hemodynamically stable and had both column right acetabular fractures, with fracture of right quadrilateral plate, superior and inferior pubic rami (Fig. 1). The patient had no known comorbidities, significant family or drug history. The patient was posted for an elective surgery.

**Fig. 2:**
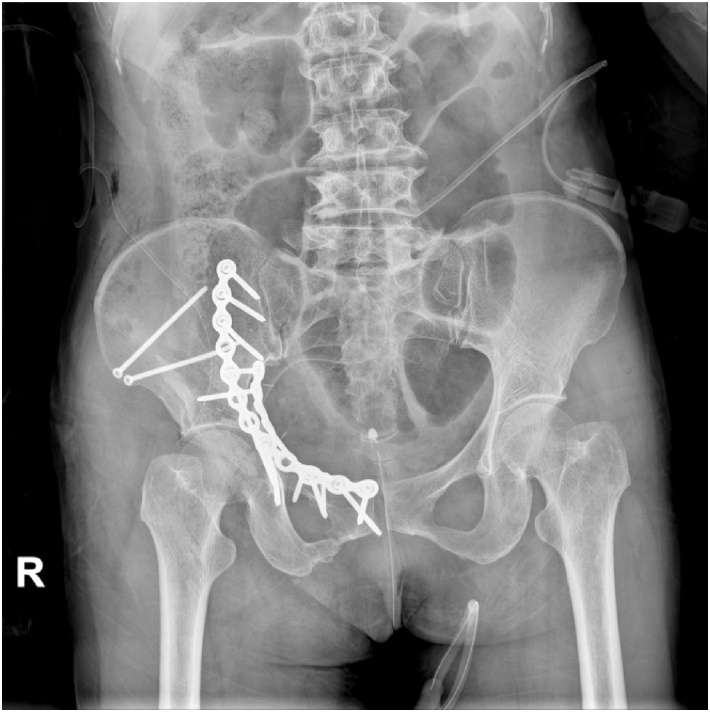
Post-operative radiograph of the pelvis showing near anatomical restoration of the fracture fragments with plates and cancellous screws.

The modified Stoppa approach with lateral window was used. The articular free fragment and anterior column reduction was done via the modified Stoppa approach. Sciatic buttress fragment and posterior column reduction was carried out using a collinear clamp and a pusher through the lateral window. Anterior column plating followed by sub-pectineal plating for fixation of the quadrilateral plate was done. In addition, two cancellous screws were passed through the iliac wing for fixation of the posterior column. The entire surgery was performed under General Anesthesia and the surgery was uneventful. Post-operative radiograph of the pelvis showed near anatomical restoration of the fracture fragments (Fig. 2).

**Fig. 3:**
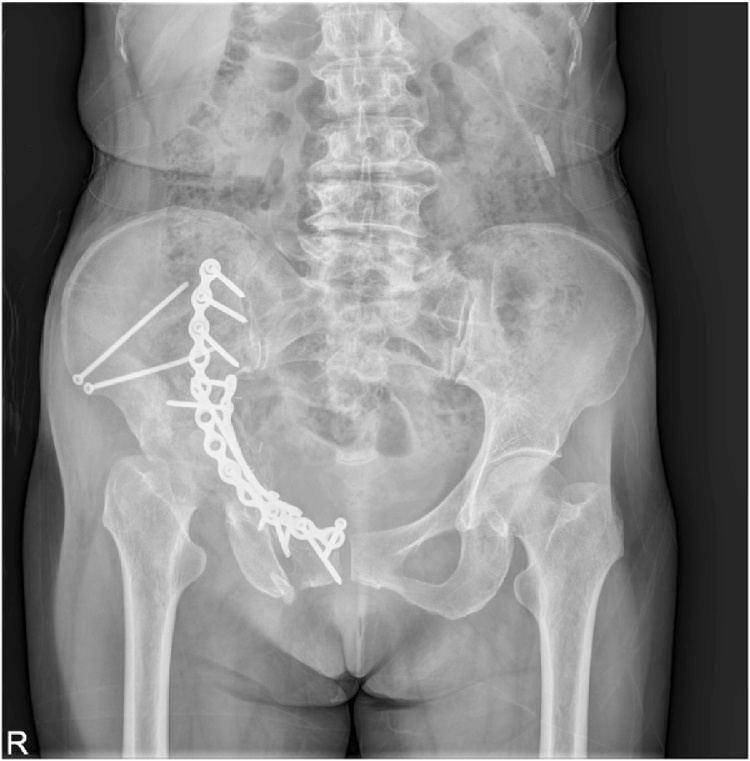
1 month post–operative radiograph of the pelvis showing normal head of the femur.

**Fig. 4:**
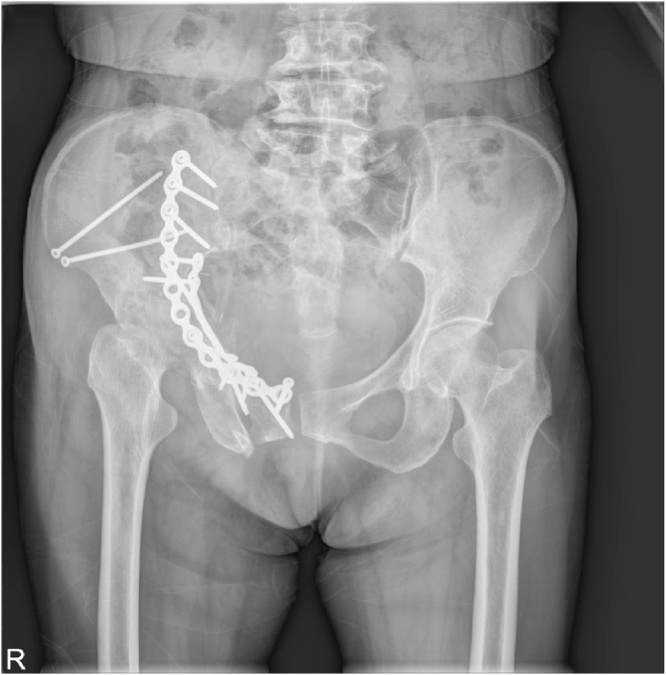
2 months post-operative radiograph of the pelvis showing signs of severe progressive destruction of the right femoral head with joint space narrowing and subchondral bone loss in the femoral head.

**Fig. 5:**
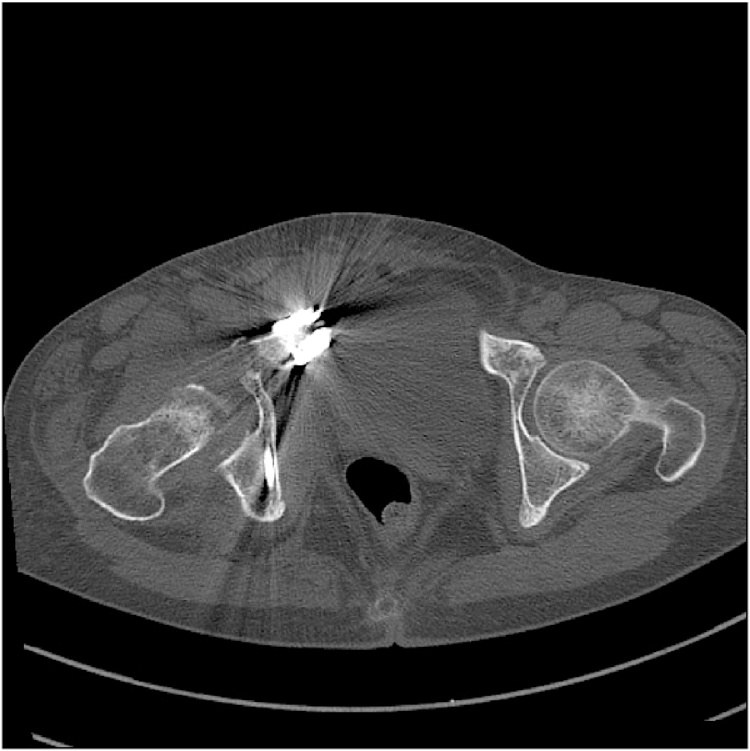
C.T. scan at 2 months showsthat the right femoral head has an anterolateral surface depressed fracture and anterosuperior subchondral insufficiency fracture.

**Fig. 6:**
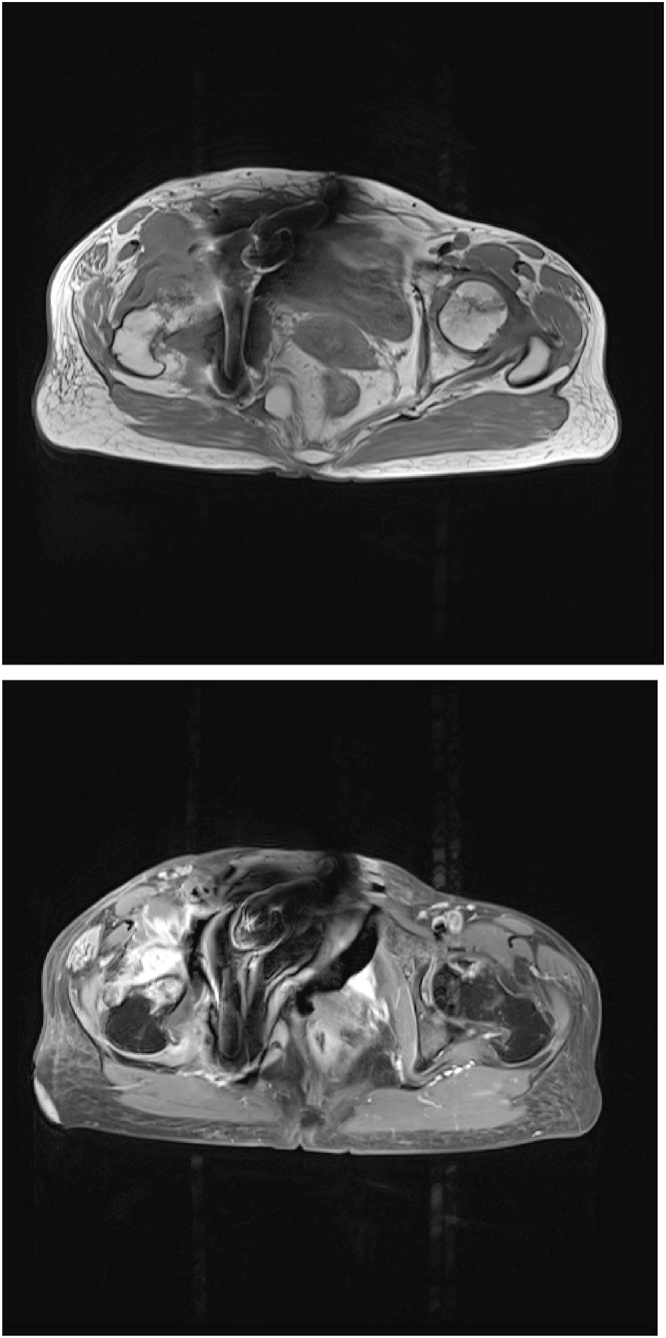
MRI T1 images showing bone marrow oedema of the femoral head with articular depression at the weight bearing surface with synovitis, synovial hypertrophy and large effusion.

Patient had regular subsequent follow-ups. One month after the surgery, the patient complained of pain in the right hip joint. The radiograph of the pelvis was repeated, which did not highlight any difference in comparison to post-operative radiogram (Fig 3). The patient was managed conservatively on analgesics. At 2 months follow-up, the patient continued with the symptoms of severe excruciating right hip pain. Further imaging studies were requested. The radiograph of the pelvis showed signs of severe progressive destruction of the right femoral head with joint space narrowing and subchondral bone loss in the femoral head (Fig. 4). C.T. pelvis revealed that the right femoral head had geographical sclerosis of the anteroposterior weight-bearing portion. In addition, the femoral head had an anterolateral surface depressed fracture and anterosuperior subchondral insufficiency fracture (Fig. 5). Magnetic Resonance Imaging (M.R.I.) of the right hip revealed an articular surface depression with bone marrow edema extending to the intertrochanteric region of the proximal femur (Fig. 6). It also displayed synovitis with large amount of effusion and synovial hypertrophy. The patient underwent Total Hip Arthroplasty. The head specimen was sent for histopathological analysis and the reports confirmed the diagnosis of rapidly destructive osteoarthritis of the hip.

## 3 Discussion

We present a case of rapid progressive hip disease, to our knowledge, the first ever reported from the Korean population. Rapid destructive osteoarthritis of hip is a rare condition of unknown etiology with rapid destruction (6–12 months) of the joint. In our case, it was within 2 months. Laquesne defined it as narrowing of the articular surface at the rate of 2 mm/year or loss of articular surface more than 50% in 1 year [13]. The disease has been reported frequently in the literature affecting the Caucasians from Europe. [1,3,[8], [9], [10]]. In addition, it is only sporadically reported in American, Japanese and Gulf countries [2,[4], [5], [6], [7],^11^].

Though the cause is not clearly understood, three factors have been postulated in the pathophysiology of the condition, which are mechanical stress, cartilage degeneration and bone response. If cartilage degeneration is slow, sclerosis and osteophytes are seen resulting in joint stability and hypertrophic osteoarthritis. On the other hand, if the rate of cartilage degeneration is fast, it leads to poor bone resorption, leading to atrophic or destructive osteoarthritis [3,11]. In addition, subchondral bone ischemia resulting in necrosis has been proposed as an important factor in pathogenesis. Other factors like Interleukin 6, 1B and serum and plasma Metalloproteinases 3 & 9 have been documented [3,4,6].

In our case, we observed the M.R.I. findings of bone marrow edema of the femoral head with the signal abnormalities extending into the intertrochanteric area. Furthermore, the femoral head depression at the weight-bearing surface with synovitis, synovial hypertrophy and large effusion was observed. All these findings were consistent with the findings reported by Boutry et al. [10]. In addition, the patient had evidence of subchondral insufficiency fracture, which has been associated to the pathogenesis of the rapid destruction of osteoarthritis joints [8].

Other potential causes that can rarely cause such severely destructive arthropathies include multicentric histiocytosis, sarcoidosis, charcoats or neuropathic arthropathy [11]. Neither the patient had any of the features of these diseases nor had any predisposing neurological problems and diabetes. Although, avascular necrosis (AVN) was possible, the M.R.I. findings of the patient was in line with the MRI findings of the rapidly progressive hip disease and confirmed our diagnosis. Thus ruling out avascular necrosis of the hip. Absence of typical clinical history, serological findings and polyarticular involvement ruled out inflammatory arthritis.

Recently, the relationship between inverted acetabular labrum and rapidly destructive hip osteoarthritis has been postulated by K. Fukui et al. [14]. According to their study, inversion of the acetabular labrum breaks the suction seal between femoral head and acetabular rim. Thus, it increases the stress on the articular cartilage leading to subchondral insufficiency fracture of the femoral head, which was noted in our case. Thus, rapidly progressive hip disease is an uncommon condition that can lead to rapid destruction of the hip joint. This condition should be taken into consideration in patients who presents with clinical and radiological features characteristic of osteoarticular destruction.

## 4 Conclusion

Rapidly progressive Hip disease is a rare finding in the Korean population as it is more commonly seen in the Caucasian population. However, orthopaedic surgeons should keep the diagnosis of it in mind in patients presenting with hip arthropathies.

## Conflict of interest

None.

## Funding source

None.

## Ethical approval

The study is exempted from ethical approval.

## Consent

Yes, written informed consent obtained from the patient.

## Author contribution

Rohan Bhimani – Study Design, Data Collection, Data analysis, and Writing the paper.

Fardeen Bhimani – Data Analysis, Writing the paper.

Preeti Singh – Data Analysis, Writing the paper.

## Registration of research studies

None.

## Guarantor

Rohan Bhimani.

## Provenance and peer review

Not commissioned, externally peer reviewed.
